# Sleep Regularity Index in Patients with Alcohol Dependence: Daytime Napping and Mood Disorders as Correlates of Interest

**DOI:** 10.3390/ijerph17010331

**Published:** 2020-01-03

**Authors:** Alyssa T. Brooks, Shravya Raju, Jennifer J. Barb, Narjis Kazmi, Subhajit Chakravorty, Michael Krumlauf, Gwenyth R. Wallen

**Affiliations:** 1 National Institutes of Health Clinical Center, 10 Center Drive, Bethesda, MD 20892, USA; sraju2494@gmail.com (S.R.); narjis.kazmi@nih.gov (N.K.); krumlaum@cc.nih.gov (M.K.); gwallen@cc.nih.gov (G.R.W.); 2 Mathematical and Statistical Computing Lab/CIT/NIH, 12 South Drive Bldg 12A Room 2001, Bethesda, MD 20892, USA; barbj@nih.gov; 3 Corporal Michael J. Crescenz VA Medical Center, Perelman School of Medicine, MIRECC, 2nd Floor, Mail stop 116, 3900 Woodland Avenue, Philadelphia, PA 19104, USA; subhajit@pennmedicine.upenn.edu

**Keywords:** alcohol use disorder, sleep disturbance, insomnia, sleep regularity, mood disorder, substance use disorder

## Abstract

Alcohol use disorder (AUD) is often accompanied by comorbid conditions, including sleep disturbances related to sleep regularity and timing. The Sleep Regularity Index (SRI) is a novel measure that assesses the probability that an individual is awake (vs. asleep) at any two time points 24 h apart. We calculated actigraphy-based SRI on 124 participants with alcohol dependence to capture the effects of changes in sleep timing and duration among patients enrolled in an inpatient alcohol treatment program. During the course of the study, the mean SRI increased between weeks 1 and 3 (75.4 to 77.8), thus indicating slightly improved sleep quality and regularity during alcohol treatment. Individuals within the bottom quartile of SRI scores at week 1 improved significantly over time. Average total SRI for individuals with no mood disorders was slightly higher than that for individuals with one or more mood disorders. Increased SRI scores were associated with lower total nap duration from week 1 to week 3. Increased SRI scores were associated with decreased mental/physical exhaustion scores from week 1 to week 3. The SRI could be a target for assessment/intervention in certain sub-groups of individuals undergoing inpatient treatment for AUD.

## 1. Introduction

Alcohol use disorder (AUD, previously alcohol abuse or dependence) is a serious disease that has far-reaching public health implications. Globally, over 283 million people are estimated to have AUD [1]. As of 2015, 16 million people in the United States are estimated to have AUD [2] AUD is often accompanied by comorbid conditions, including sleep disturbances [3], and the prevalence of sleep disturbances in populations with AUD is higher than that of the general population [4]. Sleep duration and sleep quality are often the focus of studies to examine the relationship between sleep, alcohol use, and relapse. Exploring the relationship between sleep regularity and circadian timing may be beneficial to understand the relationship between sleep and sobriety. 

Circadian rhythmicity is a universal phenomenon in all living organisms. The sleep–wake cycle is the most apparent manifestation of this rhythm; any disruption of the endogenous circadian mechanism or discordance between the internal and external environment could result in adverse sleep outcomes [5,6]. In preclinical animal studies by Thakkar and colleagues [7], alcohol is described as a “*potent somnogen*” that severely disrupts sleep homeostasis. A recent review examined sleep problems across three stages of alcohol use: binge/intoxication, withdrawal/negative affect, and preoccupation/anticipation. Sleep disturbances are ubiquitous among populations with AUD during all three stages. During the binge stage, alcohol intoxication results in shorter sleep onset latency but poorer sleep quality in the later part of sleep. Sleep problems during the withdrawal phase are highly variable. However, limited recovery from sleep disturbance during the first month of abstinence has been reported. During the long-term abstinence stage (preoccupation), various sleep issues persist such as increased sleep onset latency, more wake after sleep onset (WASO), and a decrease in slow-wave sleep (SWS) [4]. In humans, these sleep disruptions—including insomnia, sleep fragmentation, and altered sleep architecture—are especially pronounced during withdrawal and early abstinence and may persist for several years following abstinence. In addition, irregularity in sleep–wake schedules is associated with higher risk of alcohol use [8]. A recent paper by Hasler and colleagues [9] articulates the need for emerging research to delineate which specific sleep factors (including timing and regularity) as well as which circadian factors (such as chronotype and time of alcohol consumption) are related to individual responses to alcohol. Understanding sleep regularity over time in treatment-seeking individuals with AUD may provide insight to guide clinicians in identifying points of intervention.

Phillips et al. [10] developed a novel measure to assess daily variability of sleep and wake states: the Sleep Regularity Index (SRI). The SRI assesses the probability that an individual is awake (vs. asleep) at any two time points that are 24 h apart. The SRI may be an appropriate way to capture the effects of changes in sleep timing and duration, like those experienced by patients transitioning into and out of a structured inpatient treatment program. Episodes of relapse are often accompanied by sleep disturbance [11,12,13,14,15] making it difficult to describe sleep patterns with current measures that rely on defining one “major rest interval”, such as variation in the mid-sleep time point. The SRI assesses multiple rest intervals throughout the day, which may be important when considering variations in alcohol craving and ultimately relapse.

Although a relatively new measure, higher SRI scores are associated with better academic performance in college students (irregular sleep and light exposure patterns were associated with delayed circadian rhythms; [10]). Additionally, lower SRI scores are associated with increased perceived stress and depression, indicating that sleep irregularity could represent an early target for preventing cardiometabolic disease [16]. Most recently, among patients with delayed sleep–wake disorder, higher SRI scores were associated with earlier sleep and longer total sleep time [17]. According to a recent review, the correlates most commonly associated with intra-individual variability in one or more sleep/wake patterns were younger age, non-white race/ethnicity, living alone, physical health, high BMI, weight gain, depressive symptoms, stress, and evening chronotype [18].

### Purpose of Study

Although significant inter-individual variability exists among humans in sleep and circadian timing, a growing body of evidence suggests that intra-individual variability in sleep timing may exceed inter-individual variability [17]. This intra-individual variability may be further compounded by variability in alcohol consumption and severity of AUD with accompanying psychiatric co-morbidities. Given the high rates of sleep disturbance that individuals with AUD self-report at the end of their inpatient treatment program [15,19], assessing sleep regularity in this population may further refine our understanding of changes in sleep throughout inpatient treatment. Our exploratory aims are as follows: (1) To assess changes in SRI over time during inpatient alcohol treatment in order to capture intra-individual changes in sleep regularity; (2) To assess the relationship between SRI scores/changes in SRI scores over time and self-reported sleep quality and objectively-recorded measures of sleep quality and duration; and (3) to assess the relationship between SRI scores/changes in SRI scores over time and demographic/clinical variables of interest.

## 2. Materials and Methods

The study population consisted of treatment-seeking individuals with alcohol dependence originally enrolled and consented to participate in the study titled “Assessment and Treatment of People with Alcohol Drinking Problems” (NCT#00106093). The average length of inpatient stay was 31.6 days. All participants received inpatient treatment for alcohol withdrawal as well as psychosocial management. Participants were eligible for our study assessing sleep disturbance throughout inpatient treatment if they were 18 years of age or older, not enrolled onto a pharmacologic intervention study, able to understand the study, willing to complete a daily sleep diary, and willing to wear an Actiwatch to record their sleep and activity. All participants provided written informed consent and the study protocol was approved by an intramural NIH Institutional Review Board (IRB).

### 2.1. Sleep Measures

#### 2.1.1. Pittsburgh Sleep Quality Index (PSQI)

The PSQI is a validated self-rated questionnaire that measures sleep quality over a one-month period [20,21,22]. Nineteen items generate a global score with seven component scores; a global score higher than five indicates poor sleep quality. Study participants completed the PSQI on admission (at day 2) and before discharge at day 28 unless they were discharged sooner than 28 days. The focus of this analysis was the day 28 PSQI assessment as it (typically) covered most of the inpatient stay. The Cronbach’s alpha in our sample ranged from 0.70 to 0.74.

#### 2.1.2. Sleep and Symptom Diary

Daily sleep diaries were completed by participants throughout their inpatient stay (morning and night) for the primary purpose of cross-validating objective sleep data. Sleep diaries also collected additional information not captured by other assessments, such as number of nighttime awakenings, naps, and caffeinated drinks. Participants also rated mental and physical exhaustion on a 0–10 visual analog scale each evening.

#### 2.1.3. Epworth Sleepiness Scale (ESS)

The ESS assesses level of daytime sleepiness over a one-week period [23,24]. Participants rate their chances of falling asleep or dozing off in eight common everyday activities on a scale of 0–4, and a score higher than 10 is indicative of excessive daytime sleepiness. The ESS was completed weekly, with Cronbach’s alpha values in our sample ranging from 0.78 to 0.81.

#### 2.1.4. Actigraphy

“Actiwatches^®^” are small actigraphy-based wristband data loggers that record digitally-integrated measures of gross motor activity. For this study, the Philips Actiwatch-2 model was used to collect objective data on sleep quality and duration as well as daytime activity patterns. Sleep parameters assessed via actigraphy demonstrate high sensitivity and moderate accuracy for detecting sleep in populations with normal and disturbed sleep, when compared with polysomnography [25,26]. Participants wore the watch on their non-dominant wrist from enrollment in our study (day 2 of the inpatient stay) up until discharge. After device removal, data were downloaded and analyzed using the Actiware^®^ software (Respironics Actiware v.6.0.9.); Philips Respironics, Bend, Oregon. Members of the research team reviewed each individual’s file prior to analysis to screen for malfunctioning watches, corrupt data, and any other adjustment required using information from daily self-reported sleep diaries. Main outcomes from actigraphy included weekly averages of sleep efficiency, wake after sleep onset (WASO), sleep duration, time in bed, sleep onset latency, daytime activity, and SRI (calculated from minute-by-minute data which determined sleep and wake status in one-minute epochs using the Actiware algorithm).

#### 2.1.5. Calculation of the Sleep Regularity Index

We used the Python code provided by Dr. Engelhard at Duke University Medical Center to calculate the Sleep Regularity Index [10,15]. The SRI Python script was obtained from the authors from an online repository (sri_from_csv.py) downloaded from (https://github.com/mengelhard/sri). Portions of the Python script were translated and adapted to the JMP Scripting Language (JSL) in the JMP Statistical Discovery Software version 14 (SAS headquarters, Cary, NC, USA). Data cleaning was carried out by a customized JMP script to remove any personal identifying information from each file. A large cleaned data matrix with all actigraphy data was created for all participants with valid actigraphy data (N = 157) where each column was a participant’s actigraphy values and each row was a one-minute epoch for that participant. This cleaned data matrix was used as input into the adapted SRI calculation script written in JSL. The SRI was calculated based on the previous Python code with customized filters applied. In short, the SRI can be calculated by averaging all SRIs in the time interval of interest (i.e., total for all days denoted total SRI, week 1, week 2, or week 3), and then multiplying this average number by 200; then by subtracting 100 from this value. The possible range of SRI scores is from 0 to 100, with higher scores indicating higher sleep regularity. Since the SRI is a relatively new measure, there is no established normal range of scores yet. Therefore, we compared our results to SRI scores reported in previous studies using the same measure.

#### 2.1.6. Filtering of Actigraphy Data

We required that all participants in this analysis have at least seven days of actigraphy data in order to obtain an SRI for that participant. If a participant had less than seven days of data collected, they were excluded from any further analysis. We investigated how many participants would be excluded from the analysis if we removed individuals with more than two, four, and six hours of excluded data on any day in the first three weeks.

#### 2.1.7. Clinical Institute Withdrawal Assessment—Alcohol Revised (CIWA-Ar)

The CIWA is a 10-item measure that assesses the severity of alcohol withdrawal based on physical signs and symptoms [27,28,29,30]. For this inpatient population undergoing detoxification treatment, the CIWA was repeated until the scores were consistently below a range of 5–7, thereafter it was conducted on an “as indicated” basis at the discretion of healthcare team until a score of zero was obtained. For this analysis, we used the maximum CIWA score over the period of first four days of inpatient stay.

#### 2.1.8. Structured Clinical Interview for DSM-IV Axis I Disorders (SCID)

The SCID is a standard interview consisting of 11 modules that evaluate psychiatric diagnoses. These interviews were conducted by trained professionals and final Diagnostic and Statistical Manual of Mental Disorders, version IV (DSM-IV), diagnoses were determined through a consensus process involving trained psychiatrists. SCID diagnoses include mood/anxiety disorders, substance use disorders, and other disorders that are frequently co-morbid with alcohol dependence [31]. For this study, the SCID interview was conducted once during the inpatient stay.

#### 2.1.9. Timeline Followback (TLFB)

The TLFB measures alcohol drinking patterns and amounts over a fixed time, usually a period of 90 days [32]. The assessment collects drinking information using personal historical events as clues and number of items correspond to the number of days of interest [33]. For this study, the TLFB was typically administered during the first week of inpatient stay and collected drinking data for the 90 days preceding admission. Main outcomes of interest from the TLFB are average drinks per day, number of drinking days, and number of heavy drinking days.

#### 2.1.10. Comprehensive Pathological Rating Scale (CPRS)

The CPRS is a 19-item measure that assesses the severity of psychiatric symptoms and observed behaviors [34,35,36]. Two subscales from the CPRS include the Brief Scale for Anxiety (BSA) and Montgomery Asberg Depression Rating Scale (MADRS). The BSA measures pathological anxiety with or without other medical or psychological disorders and the MADRS assesses symptoms of depression. The BSA and MADRS were administered weekly throughout the inpatient stay as part of the original (“parent”) study. The Cronbach’s alpha for the BSA in our sample ranged from 0.63 to 0.69, and from 0.74 to 0.81 for the MADRS.

#### 2.1.11. Penn Alcohol Craving Scale (PACS)

The PACS is a five-item self-administered measure that assesses alcohol craving over the past week. The scale has excellent internal consistency and also has established construct, predictive and discriminant validity [37]. The PACS was administered weekly throughout the inpatient stay. The Cronbach’s alpha for the PACS in our sample was 0.94 at all time points.

#### 2.1.12. Data Analysis

Data were analyzed in several ways. First, the change of SRI between all weeks was calculated (i.e., delta SRI values: Week 3–Week 1, Week 2–Week 1, and Week 3–Week 2). In addition, the corresponding change in certain clinical variables was also calculated in order to compare these delta values to the delta SRI values. Associations between total SRIs, weekly SRIs and delta SRIs versus clinical variables and delta clinical variables of interest were calculated using Pearson correlations. The clinical variables of interest included sleep quality (PSQI at day 28), daytime sleepiness (weekly ESS scores), number of caffeinated drinks and number of night awakenings (weekly averages from diaries), naps (weekly duration in minutes from actigraphy), and number of days per week on which medications with a direct or indirect effect on sleep were consumed. In addition, we also assessed correlations between the SRI and mood- and alcohol-related variables such as anxiety and depressive symptoms (BSA and MADS), craving (PACS), and withdrawal (CIWA). The total SRIs for all participants was compared between participants with one or more mood disorders (vs. no mood disorders) using a one-way ANOVA. Also, because there was such a large range of sleep regularity at the start of the study, week 1 SRI values were stratified by the top and bottom quartiles. In order to assess longitudinal SRI changes across weeks 1, 2 and 3, the top and bottom SRI quartiles from week 1 were assessed separately in a repeated measures ANOVA calculated in the JMP v14 Statistical Discovery™ Software (SAS headquarters, Cary, NC, USA). The three delta SRI values (i.e., Week 3–Week 1, Week 2–Week 1, Week 3–Week 2) were analyzed between the top and bottom regular sleepers as described by the top and bottom week 1 SRI quartile using a two-group *t*-test.

## 3. Sample and Demographics

The mean age of the participants included in this analysis (N = 124) was 46.3 years (±9.3). The majority of the participants were male (66.1%), not currently married (82.3%), non-Hispanic (96.0%), and Black/African-American (46.0%) (Table 1). More than half of the study participants were diagnosed with a current anxiety (50.8%) or mood (56.5%) disorder at the time of admission. On average, participants reported a total PSQI score of 11.01 ± 4.41 at admission (day 2). However, on average, the sleep quality improved significantly by the end of inpatient stay (mean PSQI score = 6.46 ± 3.82). Of note, 73% of participants used medication that had a known direct or indirect effect on sleep at any point during their inpatient stay. The “direct effect” medication category included drugs primarily indicated for sleep or sleep disorders (e.g., central nervous system stimulants, antidepressants, and sedatives); those with a possible indirect effect included medications not primarily used for sleep but which have sleep/sleep problems listed as a side effect (such as benzodiazepines, analgesics, antipsychotics, and/or antihistamines).

### Participant Exclusion Filtering Results

Any participant with less than seven days of actigraphy data (due to missing or malfunctioning watches and/or corrupt data) was removed from any further analysis (N = 11). The next filtering step assessed how many hours of excluded data any participant had within the first 21 days. If any of the 146 remaining participants had four or more hours excluded on any day during the first 21 days that person was excluded (N = 22). There were a total of 43 participants who had at least two or more hours of excluded data during the first 21 days of the study and 16 participants who had at least six hours or more on any data of excluded data during the first 21 days of the study (Supplementary Materials Table S1). Based on these assessments, in order to preserve data, we excluded any participant with four hours or more of excluded data on any given day during the first 21 days of the study. Thus, SRI values were calculated for 124 participants. Mean number of days for the calculation of total SRI scores was 23.98 (±5.32).

## 4. Results

### 4.1. SRI and Assessment of Other Sleep Variables (Aims #1 and #2)

**Figure 1:**
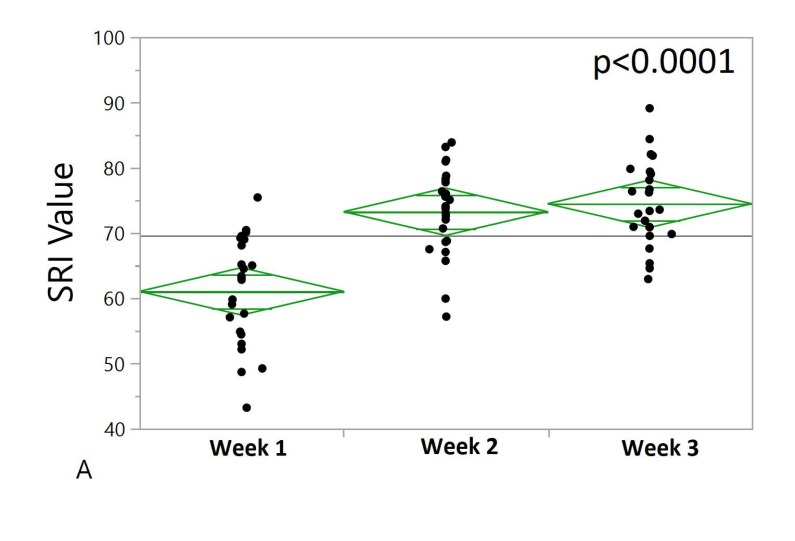

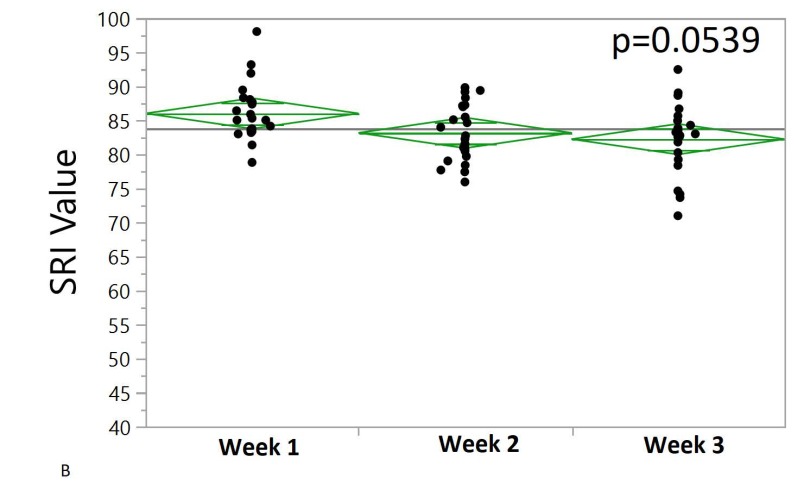
One-way plot of weekly Sleep Regularity Index (SRI) values for bottom and top quartile sleepers. Green diamonds represent confidence intervals. The center line represents the group mean. The vertical span of each diamond represents the 95% confidence interval for the mean of each group. (**A**) Bottom 25% irregular sleepers during week 1 (N = 24). Weekly SRI values (y-axis) segregated into corresponding weeks: week 1, week 2 and week 3 (x-axis). Repeated measures ANOVA shows *p* < 0.0001, N = 24. As shown by the middle line of each diamond, an average increase of SRI over three weeks is shown. The most irregular sleepers had a significant improvement in sleep regularity during the course of three weeks of inpatient treatment. (**B**) Top 25% of regular sleepers during week 1 (N = 24). Weekly SRI values (y-axis) segregated into corresponding weeks, week1, week 2 and week 3 (x-axis). Repeated measures ANOVA shows *p* = 0.0539, N = 24. There was a slight average decrease of SRI over three weeks for the top 25% SRI individuals.

Participants had a mean total SRI score of 76.95 ± 7.87, which is similar to what has been reported in other studies; mean SRI scores of 73 ± 11 reported by Phillips et al. and 71.6 ± 14.5 reported by Lunsford-Avery and colleagues. The range of SRI (total) scores in our sample was 41.46 to 90.59. The mean SRI score for week 1 was 75.72 ± 10.48 (range: 36.20–100) and it increased to 77.76 ± 10.21 (47.87–93.98) by week 3. A repeated measures ANOVA assessing the change in SRI at weeks 1, 2, and 3 among patients who had valid data at all three time points (aim #1) was non-significant (*p* = 0.065). We also stratified individuals who had data at week 1, week 2, and week 3 (N = 99) by their baseline SRI (top and bottom quartile) and repeated the ANOVA for both of those groups (Figure 1). Individuals within the bottom quartile at week 1 improved significantly over time (*p* < 0.0001). There were no significant changes in SRI over time for individuals within the top quartile.

**Figure 2:**
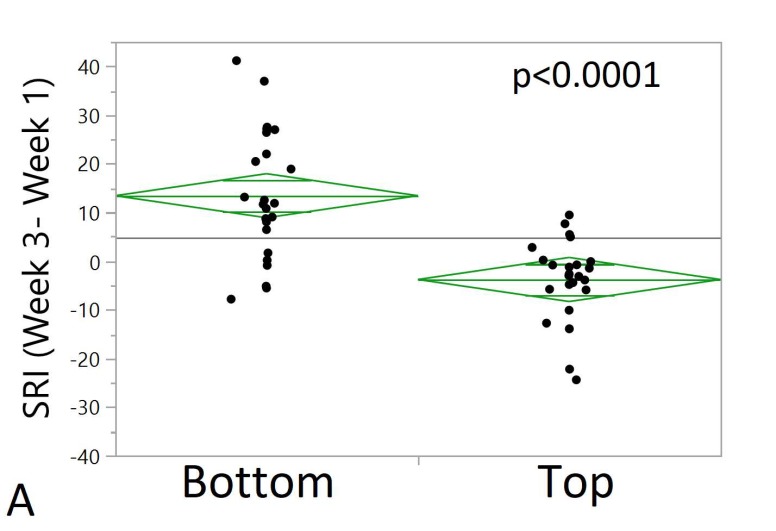

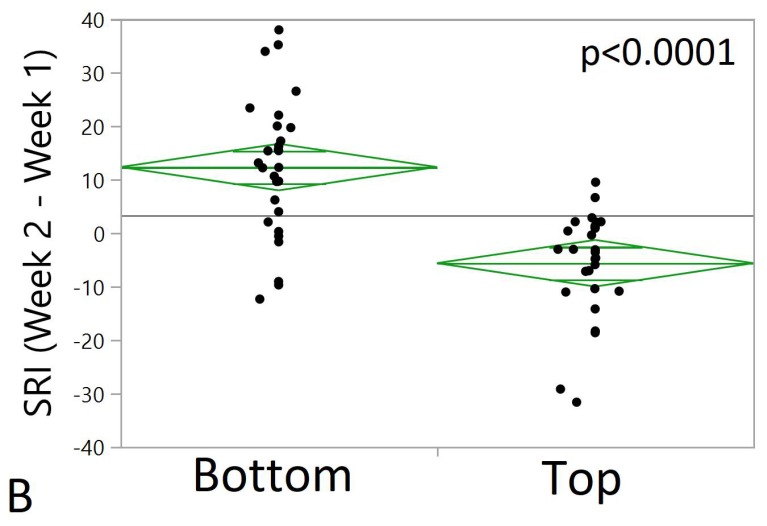

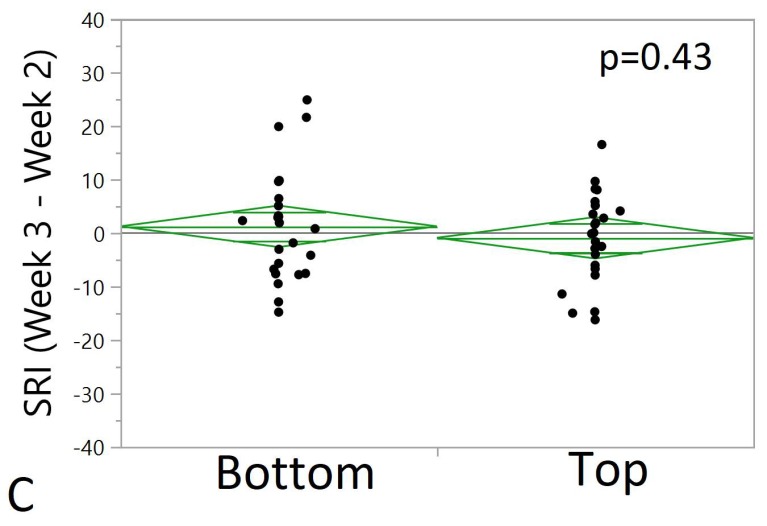
One-way plot between weekly SRI differences (Week 3–Week 1, Week 2–Week 1, Week 3–Week 2) between top and bottom baseline SRI quartiles. Green diamonds represent confidence intervals. The center line represents the group mean. The vertical span of each diamond represents the 95% confidence interval for the mean of each group. (**A**) SRI differences between week 3 and week 1 (y-axis) versus top and bottom baseline SRI quartiles (x-axis). One-way ANOVA shows a significant difference in SRI (Week 3–Week 1) between top and bottom SRI participants (*p* < 0.0001, N = 24). Change of SRI between week 3 and week 1 is significantly higher in the bottom 25% as compared to the top 25%. (**B**) SRI differences between week 2 and week 1 (y-axis) versus top and bottom baseline SRI quartiles (x-axis). One-way ANOVA shows a significant difference in SRI (Week 2–Week 1) between top and bottom SRI participants (*p* < 0.0001, N = 24). Change of SRI between week 2 and week 1 is significantly higher in the bottom 25% as compared to the top 25%. (**C**) SRI differences between week 3 and week 2 (y-axis) versus top and bottom baseline SRI quartiles (x-axis). One-way ANOVA shows no significant difference in SRI (Week 3–Week 2) between top and bottom baseline SRI quartiles (*p* = 0.43, N = 24).

We also assessed differences between week 1 and week 2, week 2 and week 3, and week 1 and week 3 between the top and bottom quartiles using one-way ANOVAs. The change of SRI between week 3 and week 1 was significantly higher in the bottom 25% as compared to the top 25%. There was a significant difference in SRI (Week 2–Week 1) between top and bottom SRI participants (*p* < 0.0001, N = 24). The change in SRI between week 2 and week 1 was significantly higher in the bottom 25% compared to the top 25%. There was no significant difference in SRI (Week 3–Week 2) between top and bottom baseline SRI quartiles (*p* = 0.43, N = 24), Figure 2. Lower PSQI (day 28) scores were significantly associated with higher SRI values during week 3 (*p* < 0.01). Higher levels of daytime activity during week 2 was significantly associated with higher SRI values (*p* < 0.01).

Table 2 shows the weekly distribution of sleep and clinical variables.

### 4.2. Correlating SRI with Clinical Variables (Aim #3)

There were no significant correlations between the total SRI (the SRI calculated across the entire inpatient stay) and sleep quality (PSQI day 28). However, there was a negative association (better sleep quality/lower total PSQI score = more regular sleep/higher SRI value), which is to be expected (*r* = −0.135, *p* = 0.24). There was a significant association between the total number of days on which “direct effect” medications on sleep were taken and weekly SRI values but only during weeks 2 (*r* = 0.93, *p* = 0.0197) and week 3 (*r* = 0.97, *p* = 0.0263).

**Figure 3:**
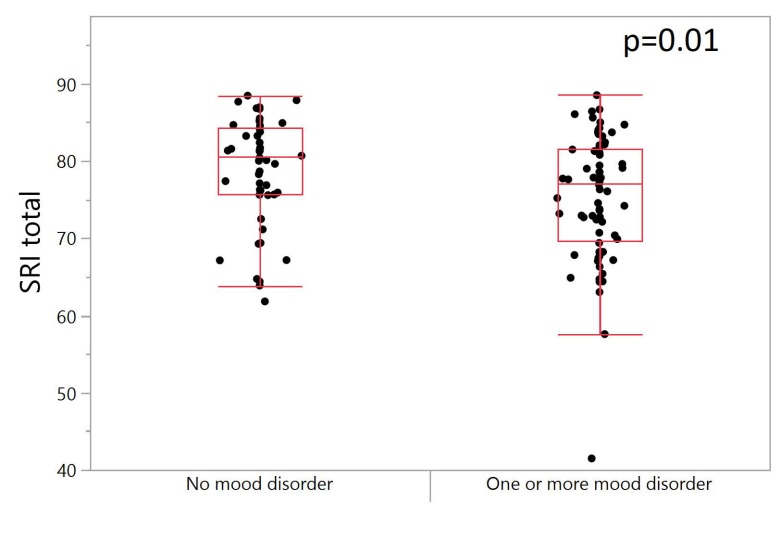
One-way plot of total SRI between participants with no mood disorder and those with one or more mood disorders. *T*-test shows a significant difference in total SRI between individuals with and without one or more mood disorder (*p* = 0.01, N = 120). Average total SRI for individuals with no mood disorders (N = 50) is slightly higher than that for individuals with one or more mood disorders (N = 70).

Participants who were diagnosed with one or more mood disorders had lower (total) sleep regularity than those without any mood disorder, and this difference was statistically significant (*p* = 0.0157), Figure 3. However, there were no significant correlations found between SRI and anxiety disorders, depressive/anxiety symptoms, demographics (race, age, gender, marital status), daytime sleepiness, severity of withdrawal symptoms, pre-admission drinking amounts and frequency, alcohol craving, self-reported number of awakenings, and caffeinated drinks.

**Figure 4:**
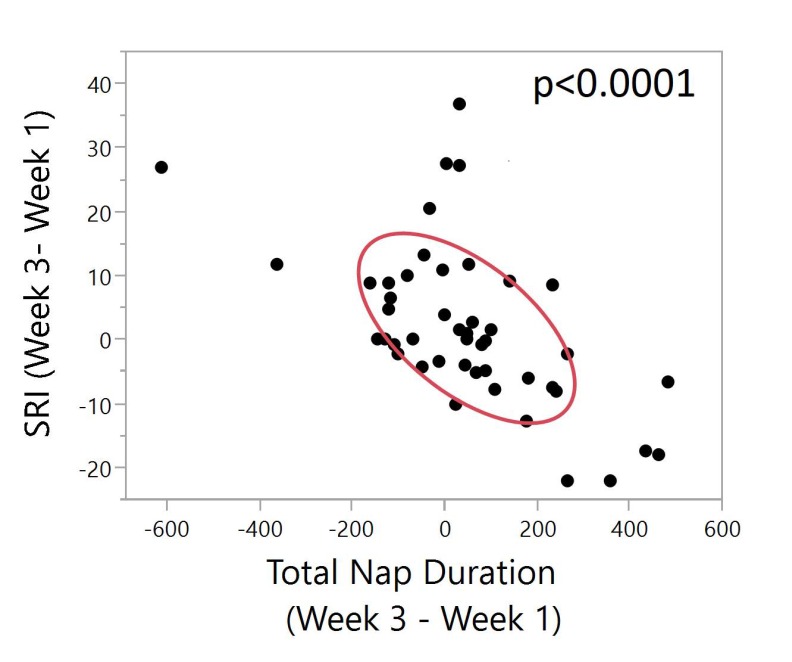
Bivariate plot of SRI (week 3–week 1) vs. total nap duration (week 3–week 1). The red ellipse shows a graphical indicator of the correlation between two differences. The red ellipsoid collapses diagonally as the correlation between them approaches either 1 or −1. The ellipsoid is more of a circle and less of a diagonal if the two variables are less correlated. The ellipse is encircling 50% of the points on the plot. A significant negative correlation is shown (Pearson, N = 46, ρ = −0.59, *p* < 0.0001) between the two differences. This can be interpreted as a higher SRI difference between week 3 and week 1 indicates improved sleep regularity between week 3 and week 1. A higher difference in total nap duration indicates increased napping time between week 3 and week 1. Significant negative correlation shows that with increased sleep regularity, there is a lower total nap duration between the two time points.

Daytime napping was assessed as total duration of naps in minutes during each week. The change of total duration of naps between week 3 and week 1 was compared to the change of SRI in week 3 versus week 1. A very strong negative correlation was found between these two delta values (*p* < 0.0001, Figure 4). Supplementary Materials Figure S1 shows the relationship between sleep regularity index and sleep duration, where SRI plots of regular and irregular sleepers also illustrate the effect of daytime napping on sleep regularity.

**Figure 5:**
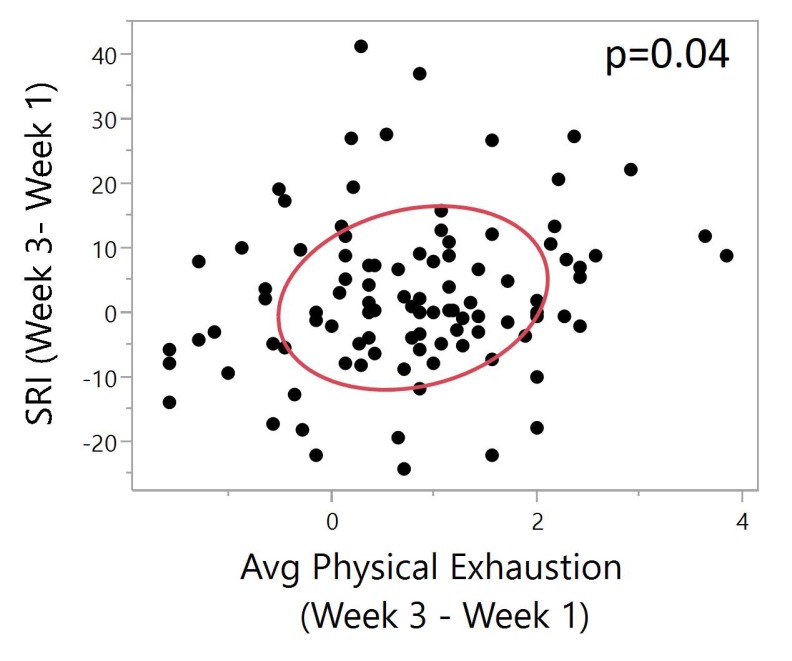
Bivariate plot of SRI (Week 3–Week 1) vs. physical exhaustion (Week 3–Week 1). The red ellipse shows a graphical indicator of the correlation between two differences. The red ellipsoid collapses diagonally as the correlation between them approaches either 1 or −1. The ellipsoid is more of a circle and less of a diagonal if the two variables are less correlated. The ellipse is encircling 50% of the points on the plot. Significant positive correlation between SRI difference and physical exhaustion difference (Pearson, N = 99, ρ = 0.20, *p* = 0.04). A higher SRI difference indicates improved sleep regularity between week 3 and week 1. A higher physical exhaustion difference indicates decreased exhaustion between week 3 and week 1. Significant correlation shows that with increased sleep regularity, there is a lower average physical exhaustion between the two time points.

There was a significant positive correlation between SRI difference and physical exhaustion difference (Pearson, N = 99, ρ = 0.24, *p* = 0.01). A higher SRI difference indicates improved sleep regularity between week 3 and week 1. A higher physical exhaustion difference indicates decreased exhaustion between week 3 and week 1. With increased sleep regularity, there is a lower average physical exhaustion between the two time points (Figure 5).

**Figure 6:**
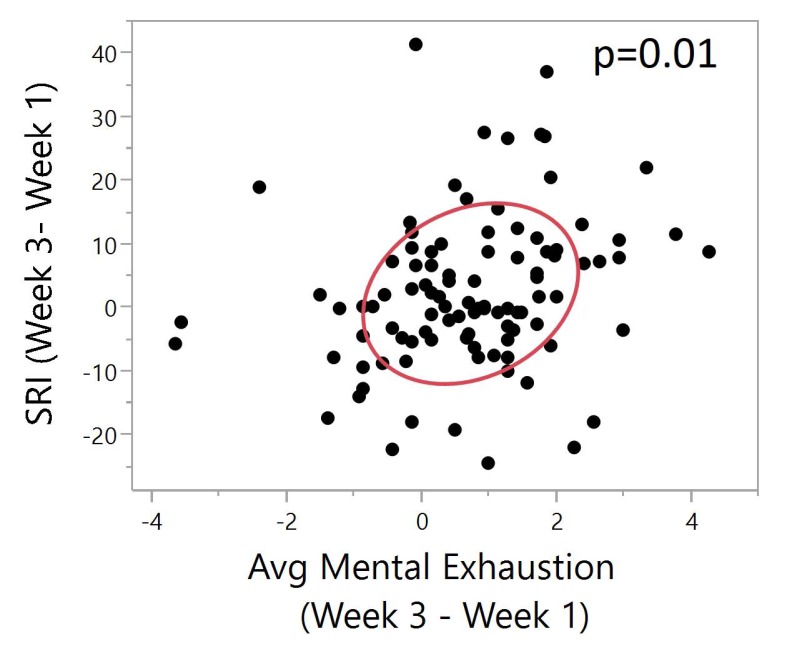
Bivariate plot of SRI (Week 3–Week 1) vs. mental exhaustion (Week 3–Week 1). The red ellipse shows a graphical indicator of the correlation between two differences. The red ellipsoid collapses diagonally as the correlation between them approaches either 1 or −1. The ellipsoid is more of a circle and less of a diagonal if the two variables are less correlated. The ellipse is encircling 50% of the points on the plot. Significant positive correlation between SRI difference and mental exhaustion difference (Pearson, N = 99, ρ = 0.24, *p* = 0.01). A higher SRI difference indicates improved sleep regularity between week 3 and week 1. A higher mental exhaustion difference indicates decreased exhaustion between week 3 and week 1. Significant correlation shows that with increased sleep regularity, there is a lower average mental exhaustion between the two time points.

There was a significant positive correlation between SRI difference and mental exhaustion difference (Pearson, N = 99, ρ = 0.24, *p* = 0.01). A higher SRI difference indicates improved sleep regularity between week 3 and week 1. A higher mental exhaustion difference indicates decreased exhaustion between week 3 and week 1. With increased sleep regularity, there is a lower average mental exhaustion between the two time points (Figure 6).

## 5. Discussion

To our knowledge, this was the first (exploratory) analysis to examine the SRI in a sample of individuals with alcohol dependence seeking inpatient treatment. Stratifying our sample based on baseline SRI scores (top and bottom 25%) allowed us to identify differences between the two groups’ trajectory over time during the inpatient stay. Sleep regularity improved slightly by the third week of inpatient stay (on average), although not significantly. However, we did find significant improvements across time in those whose baseline (week 1) SRI scores were within the bottom quartile. This could possibly be related to the structure of the inpatient alcohol treatment unit; patients were encouraged to attend scheduled group meal times and adhere to a scheduled “lights-off” time, which could have increased sleep regularity in our sample. These improvements could also be related to sustained sobriety over three weeks of inpatient treatment. We did not find any significant correlations between sleep regularity and age, gender, race or ethnicity as reported in the Bei et al. systematic review [18]. However, this could be because of the specificity of our population.

One of our most interesting findings was that participants who were diagnosed with one or more mood disorders had significantly lower sleep regularity than those without any mood disorder. However, the 95% confidence intervals between the two groups overlapped so interpretation of the findings should be considered in this context. This finding was contrary to Murray et al. [17], who found no associations between the SRI and depressive/anxiety symptoms and lower sleep regularity. However, our findings do support conclusions from a systematic review by Bei and colleagues [18] that found depressive symptoms as one of the most common correlates associated with intra-individual variability in one or more of the sleep/wake patterns. Similarly, Lunsford-Avery and colleagues found greater sleep irregularity was associated with increased perceived stress and depression in the study on the validation of the Sleep Regularity Index among older adults in a large cohort (N = 1978) study examining cardiometabolic risk data [16]. While Lunsford-Avery and colleagues suggested the SRI as a potential early target for preventing cardiometabolic disease, it remains to be seen whether the SRI could be an important cardiometabolic marker among individuals with AUD.

The total number of minutes spent napping each week were significantly negatively correlated with SRI throughout the entire study period. Additionally, improved SRI from week 1 to week 3 was associated with fewer naps. Data such as these have important implications for targeted therapies such as cognitive behavioral therapy for insomnia (CBT-I), which typically include reducing naps and/or sleep restriction as major components [38]. Such behavioral modifications could impact the SRI. We also found that decreased self-reported mental and physical exhaustion was associated with improved SRI. This finding is not surprising and may suggest assessing exhaustion as a way of identifying potential changes in SRI.

Our analysis is not without limitations. We did not measure light exposure (as in Phillips et al., 2017), which could partially explain some of our findings. Because the SRI is a relatively new measure, there exist no “normative” data for comparison. Although it is difficult to know whether the improvement in mean SRI scores from week 1 to week 3 is clinically meaningful, the clinical significance of the SRI data may be supported by positive subjective and objective sleep findings in the PSQI, SOL, sleep efficiency, and decreased WASO scores. We did not assess sleep apnea (via self-report or objective methods), which could potentially impact sleep quality and/or regularity. We did not collect data on whether or not participants had a roommate, which could have impacted their SRI. Also, we did not collect data on treatment engagement, which may have reduced craving and thus impacted sleep. We did not collect data on relapse. An analysis of the daily dosing of medications with a direct/indirect effect on sleep was beyond the scope of this analysis; however, future research should examine the effect of medication dosing and timing of administration on the SRI and other sleep indices. The decision to exclude individuals with four or more hours of missing data in any given week was somewhat arbitrary (Supplementary Materials Table S1) and thus possibly a limitation. However, we made the decision based on preserving overall “robustness” of the data (compared to using two hours or six hours as the cut off). Finally, the sample included herein was not representative of all individuals seeking inpatient treatment for alcohol dependence, thus findings should not be generalized. As with our other work in this population, the use of both objective and subjective assessment of sleep is a methodological strength.

Our analyses raised an interesting question: when assessing longitudinal datasets, should the SRI be considered as a “state” or a “trait?” In this paper, we treat it as both by examining weekly values as well as total SRI scores. Because our patients were undergoing significant changes: withdrawal/detox, adjusting to living in a hospital, and adjusting to sobriety, we believe it was important to assess (weekly) changes over time. Nevertheless, it is possible that in general, individuals’ SRI scores remain fairly stable. This could become more evident as larger, epidemiological studies begin to use the SRI as a correlate, predictor, and outcome and we begin to understand SRI values in healthy populations. Our sample was on an inpatient treatment unit with set meal times and “lights off” times—it is possible, and likely, that the variation in SRI could be much greater in a non-treatment-seeking sample without the same inpatient structured environment. Based on our findings, future research should explore whether traditional sleep hygiene interventions, such as reducing daytime naps and increasing daytime activity, can improve SRI. Future research should consider additional sensitivity analyses to identify appropriate levels of missingness when calculating the SRI. Future studies should consider predictive modeling to examine clinically meaningful outcomes such as relapse.

## 6. Conclusions

We described sleep regularity patterns of individuals with alcohol dependence in an inpatient treatment program and correlated SRI values with various demographic, clinical, and sleep-related outcomes. Mood disorders and daytime napping both correlated with SRI scores, suggesting that evaluating SRI could be a target for assessment/intervention in certain sub-groups of individuals.

## Figures and Tables

**Table 1:** Demographic, clinical and sleep variables (N = 124 *).

Characteristic	Mean (SD)
**Age**	46.3 (9.3)
	**Number (%)**
**Gender**	
Male	82 (66.1)
Female	42 (33.9)
**Race**	
Black or African American	57 (46.0)
White	56 (45.2)
American Indian/Alaska Native	1 (0.8)
Asian	3 (2.4)
More Than One Race	3 (2.4)
Unknown	4 (3.2)
**Ethnicity**	
Non-Hispanic	119 (96.0)
Hispanic	3 (2.4)
Unknown	2 (1.6)
**Marital Status**	
Divorced	14 (11.3)
Unknown	2 (1.6)
Married	20 (16.1)
Separated	10 (8.1)
Single	76 (61.3)
Widowed	2 (1.6)
Anxiety Disorders (SCID IV) **	
Yes	63 (50.8)
Mood Disorders (SCID IV) **	
Yes	70 (56.5)
**Max CIWA (Clinical Institute Withdrawal Assessment) Score (Days 1–4)**	
Mean (SD)	7.73 (5.62)
**PSQI (Pittsburgh Sleep Quality Index) (Total Score)**	
Baseline (Day 2)	11.01 (4.41)
Pre-Discharge (Day 28)	6.46 (3.82)

* Total number of participants with valid data and included in the analysis based on filtering. ** Diagnosis of one or more disorder using Structured Clinical Interview for Diagnosis-IV.

**Table 2:** Distribution of sleep and clinical variables during the inpatient stay.

Characteristic, Mean (SD)	Week 1 (N = 124)	Week 2 (N = 116)	Week 3 (N = 99)	Total (N = 124)
**SRI** (Sleep Regularity Index)	75.72 (10.48)	77.29 (10.20)	77.76 (10.21)	76.95 (7.87)
**PSQI** (Pittsburgh Sleep Quality Index)				
Baseline (day 2)				11.01 (4.41)
Before discharge (day 28)				6.46 (3.82)
**CPRS** (Comprehensive Psychopathological Rating Scale)				
BSA (Brief Scale for Anxiety)	10.97 (6.85)	6.39 (5.30)	5.49 (4.74)	
MADRS (Montgomery-Asberg Depression Rating Scale)	16.14 (9.07)	7.68 (6.44)	6.55 (6.10)	
**ESS** (Epworth Sleepiness Scale)	7.65 (4.32)	7.26 (4.26)	6.98 (4.28)	
**PACS** (Penn Alcohol Craving Scale)	11.53 (7.86)	8.56 (6.70)	7.21 (5.83)	
**Actigraphy Measures**				
Nap Duration	215.58 (207.0)	213.75 (207.22)	243.77 (239.42)	649.02 (707.61)
Sleep Efficiency	75.68 (11.23)	74.84 (12.95)	76.56 (12.35)	76.01 (10.48)
Wake after Sleep Onset	65.58 (23.99)	63.30 (24.60)	62.17 (25.93)	63.83 (21.35)
Sleep Duration	386.52 (76.48)	371.84 (74.70)	379.02 (77.75)	381.78 (67.82)
Sleep Onset Latency	15.59 (13.35)	16.44 (16.74)	15.17 (17.62)	15.18 (10.57)
Average Activity	262.68 (93.93)	179.75 (93.49)	276.11 (92.40)	272.96 (81.97)

## Supplementary Materials

The following are available online at https://www.mdpi.com/1660-4601/17/1/331/s1. Figure S1: Average sleep duration vs. SRI; Table S1: Total number of participants with excluded data within 4 weekly intervals.
