# Supplementary material for: Sleep Regularity Index in Patients with Alcohol Dependence: Daytime Napping and Mood Disorders as Correlates of Interest
Source: Int J Environ Res Public Health. 2020 Jan 3;17(1):331. doi: 10.3390/ijerph17010331 (PMC6982308; doi:10.3390/ijerph17010331)
Supplement: Supplementary file 1 [file ijerph-17-00331-s001.pdf]

Table S1. Total number of participants with excluded data within 4 weekly intervals (n = 124).

Hours Excluded	Number of participants Week 1	Number of participants Week 2	Number of participants Week 3	Number of participants Week 4
2hrsExcluded	10	24	15	37
4hrsExcluded	6	12	9	27
6hrsExcluded	4	9	7	21

These counts are not mutually exclusive in that the 6 participants in week 1 with 4 hours excluded might also be counted in week 2 with having 4 hours of excluded data as well. Week 4 data were not used for weekly analyses.

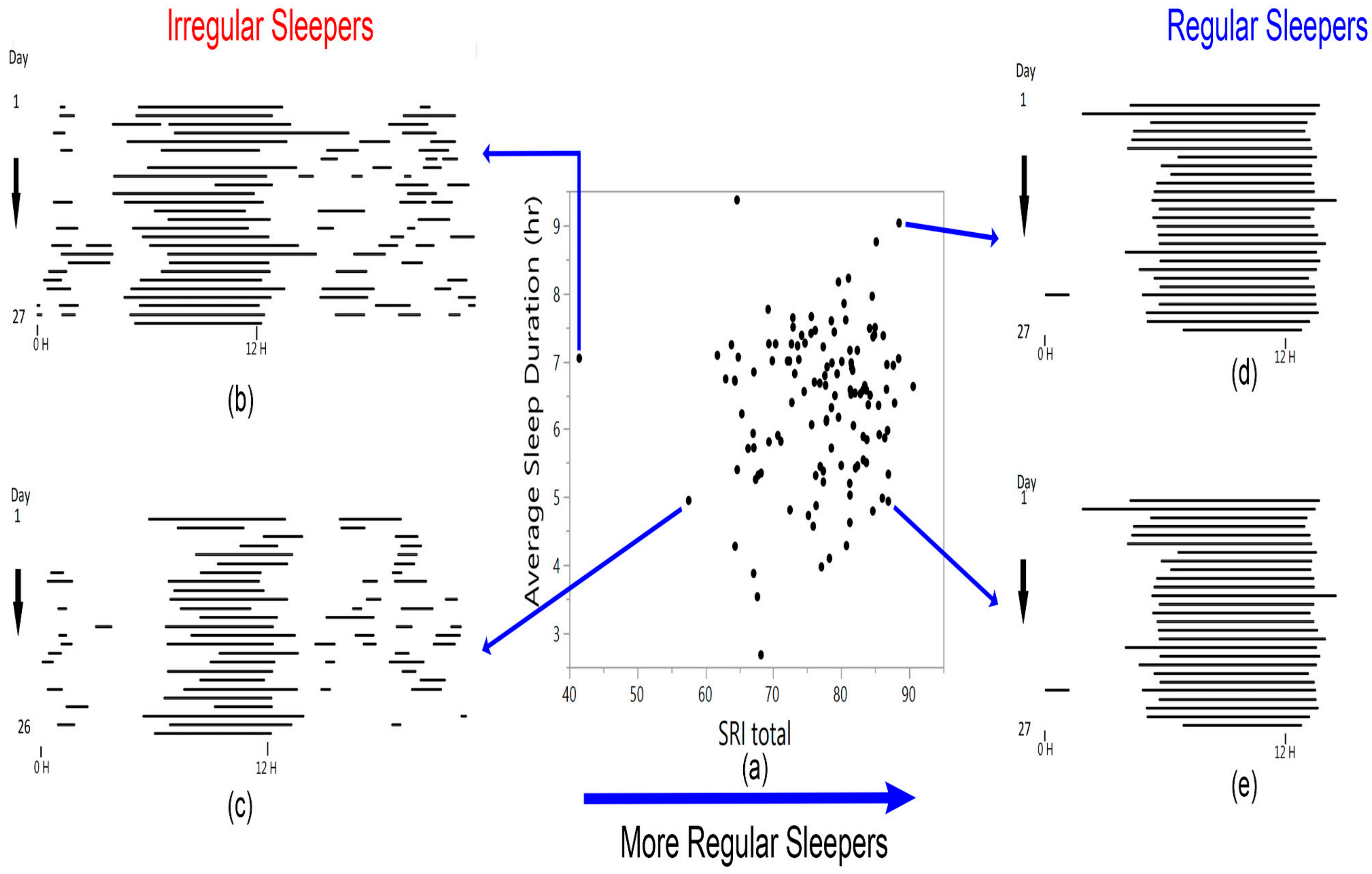

Irregular Sleepers
Regular Sleepers
Day
1
27
26
0 H
12 H
(b)
(c)
(d)
(e)
Average Sleep Duration (hr)
SRI total
(a)
More Regular Sleepers

Figure S1. Average sleep duration vs. SRI (N= 124). (a) Average sleep duration (y axis) by total SRI (x axis). Plots on either side show a schematic line plot for four individuals noted by arrows from the center plot: (b) & (c) show the sleeping patterns from two irregular sleepers (SRI: 41.46 and 57.56) but with high and low sleeping durations for 27 days (x axis) and the daily 24 hour time intervals (y axis); (d) & (e) show the sleeping patterns from two highly regular sleepers (SRI: 88.51 and 86.93) but with high and low sleeping durations for 27 days (x axis) and the daily 24 hour time intervals (y axis).
